# Lectures on the Diseases of Infancy and Childhood

**Published:** 1866-02

**Authors:** 


LECTURES ON THE DISEASES OF INFANCY AND CHILDHOOD. By CHARLES WEST, M.D., Fellow of the Royal College of Physicians; Physician to the Hospital for Sick Children. Fourth American, from the Fifth revised and enlarged English Edition. Philadelphia: HENRY C. LEA. 1866.

The merits of a book that has passed through four editions within a few years, should be well known to the profession at large. This work is a good-sized octavo volume of 654 pages, printed on good paper and fair type. The author had unusual opportunities for observation in relation to the diseases of infancy and childhood, and seems to have possessed all the requisite qualifications for improving his opportunities to good advantage. Hence, we have, in the present edition of his work, one of the very best treatises on those diseases accessible to the student and practitioner. It is worthy of a place in every physician's library.

For sale by W. B. KEEN & Co., 148 Lake St. Price $4.50.

